# Percutaneous access for the diagnosis of urothelial neoplasms: pictorial essay with anatomopathological correlation

**DOI:** 10.1590/0100-3984.2019.0091

**Published:** 2020

**Authors:** Thiago Franchi Nunes, Tiago Kojun Tibana, Rômulo Florêncio Tristão Santos, Bernardo Bacelar de Faria, Vinicius Adami Vayego Fornazari, Edson Marchiori

**Affiliations:** 1 Hospital Universitário Maria Aparecida Pedrossian da Universidade Federal de Mato Grosso do Sul (HUMAP-UFMS), Campo Grande, MS, Brazil; 2 Laboratório Scapulatempo, Campo Grande, MS, Brazil; 3 Escola Paulista de Medicina da Universidade Federal de São Paulo (EPM-Unifesp), São Paulo, SP, Brazil; 4 Universidade Federal do Rio de Janeiro (UFRJ), Rio de Janeiro, RJ, Brazil

**Keywords:** Percutaneous access, Urinary tract, Malignancy, Acesso percutâneo, Trato urinário, Malignidade

## Abstract

Urothelial carcinoma is a rare malignant neoplasm, accounting for only 5% to 7% of kidney tumors and 5% of urothelial tumors. During the management of urothelial carcinoma, anatomopathological evaluation is used for stratifying the tumors into different prognostic groups to aid in the evaluation of treatment results and to optimize the management of patients. Percutaneous image-guided biopsy is a safe and feasible procedure, with high sensitivity and accuracy rates. Although image-guided percutaneous biopsy of the urinary tract is a relatively uncommon procedure, it can be considered an option in selected cases or when traditional methods, such as the ureteroscopic technique, are not possible.

## Abstract (pt)

O carcinoma urotelial é uma neoplasia maligna rara, responsável por apenas 5% a 7% dos tumores renais e 5% dos tumores uroteliais. No manejo do carcinoma urotelial, a determinação anatomopatológica tem por objetivo estratificar os tumores em diferentes grupos prognósticos, para permitir avaliar resultados do tratamento e otimizar o gerenciamento dos pacientes. Biópsia percutânea guiada por imagem é um procedimento seguro e tecnicamente viável, com alta sensibilidade e taxa de precisão. Apesar de as biópsias percutâneas guiadas por imagem do trato urinário serem um procedimento relativamente incomum, podem ser consideradas como opção em casos selecionados ou quando os métodos tradicionais, como o a técnica ureteroscópica, não são possíveis.

## INTRODUCTION

Urothelial carcinoma is a rare malignant neoplasm, accounting for only 5% to 7% of kidney tumors and 5% of urothelial tumors. Although radical surgery is the gold standard treatment for patients with urothelial carcinoma, endoscopic advances have resulted in favorable outcomes after treatment and renal preservation in selected cases, even in patients with a normal contralateral kidney^(1,2)^. As the careful selection of patients for treatment is crucial, obtaining a satisfactory sample for diagnosis is an essential step in determining the treatment and prognosis of patients with urothelial carcinoma^(3)^.

Biopsy is an integral component for the evaluation of potentially malignant lesions of the ureter and other malignancies of the upper urinary tract. When indicated, biopsy is usually performed via ureteroscopy. However, ureteroscopic biopsy may not be possible in patients with high-risk comorbidities, as it is invasive and requires general anesthesia and the insertion of ureteral catheters. Thus, although ureteroscopy remains the gold standard, it can be technically challenging and is associated with significant rates of false-negative results, depending on the morphological characteristics of the lesion^(4)^. Several image-guided interventional techniques have been evaluated in Brazilian radiology studies^(5-9)^.

Percutaneous biopsy can be performed in selected cases when the target segment of the ureter or renal pelvis cannot be accessed via ureteroscopy, is predominantly exophytic and non-endoluminal, or showed inconclusive results in previous samples.

## ULTRASOUND-GUIDED BIOPSY (TRANSABDOMINAL AND ENDOCAVITARY)

**Figure 1:**
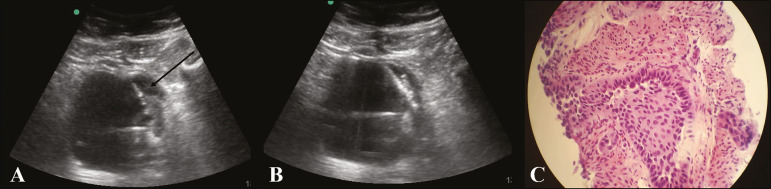
**A:** Ultrasound of the bladder showing a 17-G × 13-cm coaxial needle (arrow) positioned adjacent to the vegetating lesion on the left-side wall of the bladder. **B:** Percutaneous biopsy using an 18-G × 16-cm tru-cut needle. **C:** Anatomopathological examination revealed invasive urothelial carcinoma.

Ultrasound appears to have several advantages as a guidance method. It is commonly available and does not involve ionizing radiation, and the device is portable and provides images in real time (Figure 1). Unfortunately, not all small kidney tumors can be visualized on ultrasound, and adjacent structures and organs may not be differentiated properly, unlike that observed using computed tomography (CT). Furthermore, gaseous and bony structures may impair visualization^(10)^. However, the needle can be directed towards solid components in the mass, and the location can be confirmed at the time of the biopsy, thereby obtaining a more precise positioning of the needle and a better fragment of the lesion^(10)^.

## CT-GUIDED BIOPSY

**Figure 2:**
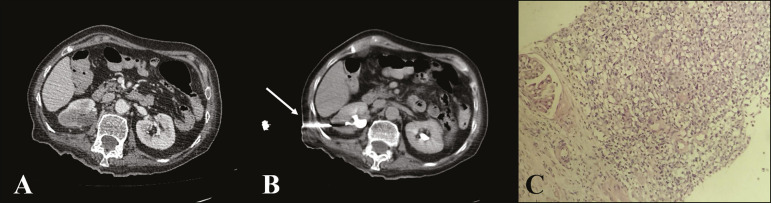
**A:** CT scan of the abdomen, axial section, showing an ill-defined urothelial lesion in the middle third of the right kidney. **B:** Performance of the CT-guided biopsy (arrow) using the coaxial technique after injection of the contrast agent in the excretory phase. **C:** Anatomopathological examination revealed invasive urothelial carcinoma.

CT-guided percutaneous biopsy of ureteral lesions and collecting system is a useful alternative to obtain samples of suspicious lesions in challenging clinical situations (Figure 2). Common clinical scenarios appropriate for a CT-guided percutaneous approach include cases of ileal urinary ducts, unfavorable anatomy, inability to perform ureteroscopic access, inability to perform the ultrasound-guided procedure, or unsuccessful ureteroscopic biopsy in cases with high clinical suspicion of malignancy^(11)^.

## FLUOROSCOPY-GUIDED FORCEPS BIOPSY

**Figure 3:**
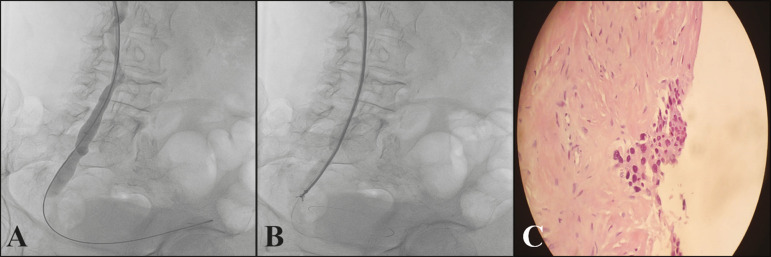
**A:** Urethrography showing an obstructive lesion in the right distal ureter and crossing of the obstructive site using a hydrophilic guidewire up to the bladder. **B:** A 7-F × 45-cm sheath positioned up to the region close to the obstructive site, and performance of forceps biopsy. **C:** Pathological examination revealed well-differentiated urothelial neoplasia.

Forceps are very effective for obtaining tissues of malignant biliary stenoses^(12,13)^. The tool is composed of a sheath and forceps, which are advanced alongside a 0.035 guidewire as needed. This technique allows performing biopsy of the stenosis through the sheath, leaving a 0.035 thread to preserve access to the lesion (Figure 3). This is also advantageous when the distal wire needs to be correctly positioned for subsequent procedures. Despite its reported success, very few studies have addressed the use of forceps and its techniques to obtain samples from non-biliary regions^(14)^. Moreover, the use of forceps is a relatively safe and effective technique that makes histology-guided oncological treatment possible^(15)^.

## BIOPSY WITH ASSOCIATED TECHNIQUES

**Figure 4:**
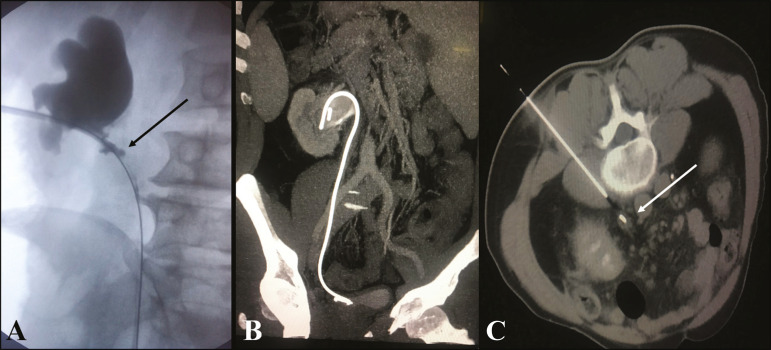
**A:** Pyelography performed before percutaneous insertion of the double J catheter showing stenosis of the proximal ureter with parietal irregularities (arrow). **B:** Post-contrast coronal abdominal CT scan, MIP reconstruction, showing the properly positioned double J catheter. **C:** CT-guided percutaneous biopsy with posterior access and coaxial technique showing ureter thickening with maintenance of the double J catheter for ureteral protection. Anatomical and pathological examination revealed an inflammatory pseudotumor of the ureter.

**Figure 5:**
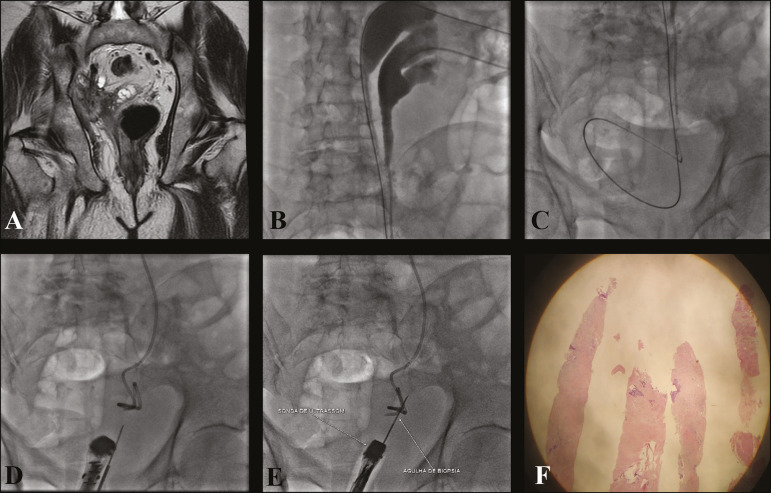
**A:** T1-weighted magnetic resonance imaging of the pelvis showing marked hydronephrosis of the right kidney, along with an extraperitoneal pelvic lesion in the distal ureter and signs of invasion of the bladder, seminal vesicle, and prostate. **B:** With the patient in the supine position, pyelography was performed before the procedure that confirmed complete ureteral duplication. **C:** Percutaneous insertion of a double J catheter (upper cup) and nephrostomy (lower cup). **D,E:** Endorectal ultrasound and fluoroscopy-guided percutaneous biopsy. **F:** Anatomopathological report revealed poorly differentiated urothelial neoplasia.

Current imaging modalities allow performing biopsies of focal lesions in several deep abdominal and pelvic organs, thereby helping in obtaining a safe and effective diagnosis^(16,17)^. In situations where bladder tumors are not easily accessed via biopsy or cystoscopy, alternative methods for the acquisition of tissue samples, such as CT, ultrasound, or fluoroscopy, can be used alone or together to perform a percutaneous biopsy (Figures 4 and 5).

## CONCLUSION

Image-guided percutaneous biopsy of the upper urinary tract collecting system appears to be a safe and effective alternative in selected patients who underwent unsuccessful ureteroscopy or who are inadequate candidates for this procedure. In selected patients who do not have a double J catheter, insertion can be performed using the percutaneous technique^(18)^ before biopsy.
